# Prevalence, infectivity and correlates of hepatitis B virus infection among pregnant women in a rural district of the Far North Region of Cameroon

**DOI:** 10.1186/s12889-015-1806-2

**Published:** 2015-05-02

**Authors:** Jean Jacques N Noubiap, Jobert Richie N Nansseu, Shalom Tchokfe Ndoula, Jean Joel R Bigna, Ahmadou M Jingi, Joël Fokom-Domgue

**Affiliations:** Department of Medicine, Groote Schuur Hospital and University of Cape Town, Cape Town, South Africa; Medical Diagnostic Center, PO Box 6230, Yaoundé, Cameroon; Department of Public Health, Faculty of Medicine and Biomedical Sciences, University of Yaoundé I, Yaoundé, Cameroon; Guidiguis Health District, Guidiguis, Cameroon; Goulfey District Hospital, Goulfey, Cameroon; Department of Internal Medicine and Specialties, Faculty of Medicine and Biomedical Sciences, University of Yaoundé I, Yaoundé, Cameroon; Department of Obstetrics and Gynecology, Faculty of Medicine and Biomedical Sciences, University of Yaoundé I, Yaoundé, Cameroon

**Keywords:** Hepatitis B virus, HBsAg, HBeAg, Pregnancy, Cameroon

## Abstract

**Background:**

Epidemiological data on hepatitis B virus (HBV) infection among pregnant women in Cameroon are very scarce, especially in the rural milieu. The purpose of this study was to determine the prevalence and factors associated with HBV infection, and the infectivity of rural pregnant women in the Far North Region of Cameroon.

**Methods:**

A cross-sectional study was conducted in three rural health facilities of the Guidiguis health district between December 2013 and March 2014. We consecutively recruited 325 pregnant women attending antenatal consultations. A pretested questionnaire was used to collect socio-demographic data and factors associated with HBV infection. The presence of hepatitis B surface antigen (HBsAg), hepatitis B e antigen (HBeAg) and human immunodeficiency virus (HIV) were determined using commercial test strips. Regression analyses were used to assess correlates of HBV infection.

**Results:**

The mean age was 24.4 (SD5.6) years. Most women were married (97.2%) and housewives (96.4%), with less than secondary education level (80%). Only 4 women (1.2%) had been vaccinated against HBV. Thirty-three women (10.2%) were HBsAg-positive, of whom 4 (12.1%) were positive to HBeAg. The prevalence of HIV infection was 2.5% (8/325). Overall, 5 (1.5%) women were co-infected with HIV and HBV. Independent correlates of HBV infection included history of blood transfusion (adjusted odd ratio 12.59, 95% CI 1.46-108.89; p = 0.021) and concurrent infection by HIV (adjusted odd ratio 22.53, 95% CI 4.76-106.71; p < 0.0001).

**Conclusion:**

The prevalence of HBV infection among pregnant women in this rural milieu is high. History of blood transfusion and HIV infection are highly associated with HBV infection. The relative low rate of women positive to both HBsAg and HBeAg suggests that perinatal transmission of HBV might not be the prevailing mode of HBV transmission in this area.

## Background

Hepatitis B virus (HBV) infection is a major health problem worldwide owing to its high prevalence and significant morbidity and mortality. There are about 2 billion people who have been exposed to HBV worldwide, and about 360 million chronic carriers [1]. More than 780,000 people die annually as a result of HBV-related liver diseases including chronic hepatitis, cirrhosis and hepatocellular carcinoma, making HBV infection the 10^th^ leading cause of death globally [1,2]. The burden of HBV is highest in Africa where approximately 65 million chronically infected individuals live, with prevalence rates ranging from less than 7% to more than 20% in some countries [3,4].

Mother-to-child transmission (MTCT) of HBV is responsible for more than one third of chronic HBV infections globally [5]. Indeed, perinatal transmission seems to be predominant in high-prevalence areas such as sub-Saharan African countries [6]. Children born to mothers positive for hepatitis B surface antigen (HBsAg) and hepatitis B e antigen (HBeAg) have a 70-90% likelihood of perinatal acquisition of HBV infection, and up to 90% of perinatal infections evolve towards chronicity compared to nearly 5% of adult infections [7]. Prevention of perinatal transmission of HBV is therefore crucial to tackle the burden of the disease in high endemic sub-Saharan African areas. Effective strategies for reducing the incidence of chronic infections include maternal screening combined with post-exposure prophylaxis consisting of HBV vaccination immediately after delivery in all children born to HBsAg positive mothers, ideally with immunoglobulin prophylaxis [8].

Chronic HBV is highly endemic (prevalence ≥ 8%) in Cameroon, a central African country [9]. However, data on the epidemiology of HBV infection in this country are scarce, and those available are mostly from urban areas. In recent studies, Noubiap et *al*. and Fouelifack Ymele et *al*. reported HBsAg prevalence as high as 10.1% and 12.1% among healthy blood donors, respectively [10,11]. Frambo et *al*. and Fomulu et *al*. estimated the prevalence of HBsAg in pregnancy at 9.7% and 7.7%, respectively [12,13]. Despite the perinatal burden of HBV, pregnant women in Cameroon are not routinely screened in most health facilities. Moreover, routine vaccination of infants against HBV within the national Expanded Program on Immunization starts at 6 weeks [12].

This study aimed to determine the prevalence and factors associated with HBV infection, and the infectivity of rural pregnant women in the Far North region of Cameroon.

## Methods

### Ethical considerations

This study was performed in accordance with the guidelines of the Helsinki Declaration and was approved by the Regional Office of the Ministry of Public Health (MOH) for the Far North region, acting as Ethics Committee. Written informed consent was obtained from all participants. HIV- and HBV-infected women were referred to health officers for appropriate management according to local protocols.

### Setting and study population

This was a cross-sectional study conducted between December 2013 and March 2014 in three rural health facilities at Toulom, Dziguilao and Guidiguis. These villages are part of the Guidiguis health district which covers a population of 150,963 inhabitants and is located 135 km south-east of Maroua, the administrative headquarter of the Far North Region of Cameroon.

The study population comprised all pregnant women who attended antenatal care in the selected health centers during the study period. We used the Window Program for Epidemiologist version 11.25 to calculate the minimal required sample size. Assuming a 9.7% prevalence of HBsAg among pregnant women as found in a previous study in Cameroon [12], a 5% margin of error and a 95% confidence interval, the minimal sample size of the study population was 135 participants. We consecutively recruited all pregnant women consenting to participate in the study until we reached a target sample size of 325 in the three sites.

### Data collection and laboratory investigation

A structured pretested questionnaire was used to collect socio-demographic information and data on risk factors for HBV infection among participants. Upon completion of the questionnaire, 5 ml of venous blood was aseptically collected by venipuncture into an Ethylene Di-amine Tetra-acetic Acid (EDTA) tube. The plasma obtained from each sample was tested for the presence of HBsAg using a commercial test strip, the DiaSpot One Step Hepatitis B test (DiaSpot Diagnostics, USA), according to the manufacturer’s instructions. All samples tested positive were retested for confirmation using the same kit. There were no discordant results. Samples confirmed positive for HBsAg were further tested for HBeAg using a commercial test strip, the DiaSpot One Step HBeAg test (DiaSpot Diagnostics, USA). We also tested all blood samples for HIV according to the national screening algorithm. Accordingly, samples were first screened with an anti-HIV antibody rapid test, the Determine HIV-1/2 (Abbott Laboratories, IL, USA), and positive samples were retested with the OraQuick ADVANCE® Rapid HIV-1/2 Antibody Test (OraSure Technologies Inc, Pennsylvania, USA). Samples giving discrepant HIV results were sent for confirmation by enzyme immunoassay.

### Data analysis

Data were coded, entered and analyzed using IBM SPSS for Windows, version 20.0 (IBM Corp., Armonk New York, USA). Results are presented as counts (proportions) and means with standard deviations (SD). Odds ratios (OR) with their 95% confidence intervals (CI) served to investigate the influence of various factors on the occurrence of HBV infection. They were calculated by both univariate and multivariate logistic regression analyses while adjusting for potential confounders. We included in the multivariate model the age and all variables with a *p* value ≤ 0.25 in univariate analyses. For this purpose, we categorized some variables into two groups: age (≤24 and > 24 years); marital status (married and not married); number of pregnancies (≤2 and > 2); past history of abortion (“yes” or “no”). Educational level was classified as ‘low’ for women having a primary level of education or less, and ‘high’ for those with a secondary level of education or more. Profession was categorized as ‘no job’ for housewives and ‘active’ for salaried employees or traders. A *p* value < 0.05 was set as statistically significant.

## Results

We recruited 325 pregnant women, the general background of whom is displayed in Table 1. Age ranged from 15 to 40 years with a mean of 24.4 (SD 5.6) years. Most of our respondents (80%) were not beyond the primary school and 313 (96.4%) women were housewives. Christians were more represented (47.7%), more so as married women (97.2%) (Table 1).

**Table 1:** **Demographics of pregnant women attending antenatal care in in the Guidiguis health district, December 2013-March 2014**

**Variable**	**Number (N = 325)**	**Percentage (%)**
Age		
≤24 years	172	52.9
>24 years	153	47.1
Religion		
Muslim	94	28.9
Christian	155	47.7
Other	76	23.4
Marital status		
Single	8	2.5
Married	316	97.2
Widowed	1	0.3
Educational level		
Never went to school	127	39.1
Primary	133	40.9
Secondary	61	18.8
University	4	1.2
Ethnic group		
Peuhl	94	28.9
Tupuri	206	63.4
Musdang	16	4.9
Other	9	2.8
Profession		
Housewife	313	96.4
Salaried employee	6	1.8
Trader	6	1.8

Risk factors for HBV infection are presented in Table 2. The main factors reported were: history of ritual scarifications (19.2%), family history of known HBV infection (6.5%), dental care (3.1%), and previous surgery (2.2%). Only 4 women (1.2%) had been vaccinated against HBV. The total number of pregnancies per woman ranged from 1 to 16 with a mean of 3.6 (SD 2.6) pregnancies, and 42 women (12.9%) had a past history of abortion.

**Table 2:** **Risk factors of HBV infection and markers of HBV infectivity among pregnant women attending antenatal care in in the Guidiguis health district, December 2013-March 2014**

**Characteristic**	**Number (N = 325)**	**Percentage (%)**
Risk factors		
Previous blood transfusion : Yes	4	1.2
Ever been scarified : Yes	59	19.2
Underwent excision : Yes	3	0.9
Ever been operated : Yes	7	2.2
Previous dental care : Yes	10	3.1
Family history of HBV infection : Yes	21	6.5
Vaccinated against HBV: No	4	1.2
History of HBV infection during previous pregnancies: Yes	1	0.3
Number of pregnancies ever had		
≤2	135	41.5
≥3	190	58.5
Past history of abortion		
No	283	87.1
Yes	42	12.9
Resuts of tests		
HIV serology		
Negative	317	97.5
Positive	8	2.5
HBs Antigen		
Negative	292	89.8
Positive	33	10.2
HBe Antigen (N = 33)		
Negative	29	87.9
Positive	4	12.1

The prevalence of HBV infection was 10.2% (33/325). Among women positive to HBsAg, only 4 (12.1%) were also positive to HBeAg (Table 2). Furthermore, 8 (2.5%) women were HIV positive. Overall, 5 (1.5%) pregnant women were co-infected with HIV and HBV. Factors independently associated with HBV infection included previous history of blood transfusion (adjusted OR 12.59, 95% CI 1.46-108.89; p = 0.021) and co-infection with HIV (adjusted OR 22.53, 95% CI 4.76-106.71; p < 0.0001). The other factors assessed did not influence the occurence of HBV infection among our participants (Table 3).

**Table 3:** **Crude and adjusted correlates of HBV infection among pregnant women attending antenatal care in the Guidiguis health district, December 2013-March 2014**

**Variable**	**Crude odds ratio**	**95% CI**	***p*** **value**	**Adjusted odds ratio** ^*^	**95% CI**	***p*** **value**
Age						
≤24 years	0.81	0.40 – 1.69	0.590	0.50	0.19 – 1.30	0.115
≥25 years	1			1		
Religion						
Muslim	0.94	0.30 – 2.92	0.913			
Christian	1.73	0.66 – 4.50	0.262			
Other	1					
Marital status						
Married	0.79	0.09 – 6.62	0.827			
Not married	1					
Educational level						
Low	0.92	0.38 – 2.22	0.854			
High	1					
Profession						
No job	1.25	0.16 – 10.02	0.832			
Active	1					
Previous transfusion						
No	1			1		
Yes	9.36	1.27 – 68.76	0.028	12.59	1.46 – 108.89	0.021^**^
Ever been scarified						
No	1					
Yes	1.51	0.65 – 3.54	0.341			
Underwent excision						
No						
Yes	1.11	1.07 – 1.16	0.725			
Ever been operated						
No						
Yes	1.12	1.08 – 1.16	0.469			
Previous dental care						
No	1					
Yes	0.98	0.12 – 8.01	0.987			
Family history of HBV						
No	1					
Yes	0.43	0.55 – 3.27	0.411			
Vaccinated against HBV						
No	1					
Yes	1.11	1.07 - 1.16	0.650			
Number of pregnancies						
≤2	1.57	0.76 – 3.22	0.223	2.60	0.97 – 6.95	0.058
>2	1			1		
Past history of abortion						
No	2.46	0.57 – 10.68	0.229	2.55	0.48 – 13.13	0.264
Yes	1			1		
Concurrent HIV infection						
No	1			1		
Yes	17.20	3.90 – 75.80	<0.0001	22.53	4.76 – 106.71	<0.0001^**^

CI: confidence interval.

^*^Adjusted for age, history of blood transfusion, number of pregnancies, number of abortions and HIV status.

^**^ *p* value < 0.05.

## Discussion

We investigated the seroprevalence and factors associated with HBV infection in a rural area of the Far North region of Cameroon. The information gathered by this work may contribute to improve knowledge on HBV infection epidemiology in pregnant women in that region, and to inform local and national antenatal HBV screening and infant immunization policies.

We found that 10.2% of pregnant women were infected with HBV in this rural area. According to WHO, the prevalence of HBV among pregnant women in this study is classified as high (≥8%) [9]. Our study adds to the various endemicity data reported among pregnant women in sub-Saharan African countries: 6.5% in Congo [14], 9.3% in Kenya [15], 9.5% in Gabon [16], 10.7% in Burkina Faso [17], 10.9 % in Mauritania [18], 12.6% in Ghana [19], 13.8% in Senegal [20], and 25% in Zimbabwe [21]. Prevalence of HBV in this study conforms with the 9.7% prevalence rate found among pregnant women in a semi-urban setting in Buea (South West region of Cameroon) [12], but is greater than the 7.7% and 7.85% reported in two studies conducted in urban health facilities in Yaoundé, the capital city of Cameroon [13,22]. Contrariwise, HBV prevalence in this study was about half the 20.4% prevalence reported among pregnant women in Tokombéré, another rural district in the Far North region of Cameroon [23]. The HBV prevalence found in Tokombéré is surprisingly high, probably the highest reported nationwide. Although our study and the just-mentioned one were conducted in rural settings of the Far North region of the country, the striking difference between the two prevalences may be explained by the fact that Tokombéré is a village situated near politically unstable areas of Chad and Nigeria, both presenting a significant rate of emigration and seemingly high HBV prevalence (7-30%) [23,24].

MTCT of HBV is reported to be responsible for more than one third of chronic HBV infections globally [5], and perinatal infection is assumed to be a major mode of transmission in high-prevalence areas like most sub-Saharan African countries [6]. HBeAg positivity is associated with a high risk of perinatal transmission of HBV as children born to mothers positive to both HBsAg and HBeAg have a 70-90% chance of perinatal acquisition of HBV infection [7]. Compared to our results, the study conducted in Tokombéré, reported a high rate of HBeAg and elevated viremia among HBsAg-positives (22.7%), suggesting a high rate of HBV perinatal transmission [23]. Another study conducted in an urban setting in Cameroon also found a high rate of HBV infectivity, with 28% of HBsAg-positive pregnant women being HBeAg-positive [13]. These findings are consistent with the results of a systematic literature review exploring the risk of perinatal HBV transmission based on HBeAg prevalence estimates from all regions of the world. It revealed that in 2005, 22.7% to 29.9% of HBsAg-positive women of reproductive age in Central sub-Saharan Africa were HBeAg-positive [25]. Our study found a lower rate of HBV infectivity, with a prevalence of HBeAg among HBsAg-positive women of 12.1%. The overall prevalence of HBeAg in our sample was 1.2%. This suggests that the probability of HBV MTCT was high only in a small proportion of our study population. Another study in Yaoundé found that none of the HBsAg-positive pregnant women with known HIV status were positive to HBeAg, suggesting that perinatal transmission plays a minor role in HBV burden in Cameroon [22]. Interestingly, current recommendation of the national immunization program of Cameroon to vaccinate all newborn at 6, 10 and 14 weeks of age is probably based on the evidence that horizontal transmission during early childhood could be the most common mechanism of HBV infection, as reported in other sub-Saharan African countries [26-28]. The conflicting data on HBV infectivity among pregnant women and MTCT in sub-Saharan African countries call for further investigations.

The prevalence of HIV infection and HIV/HBV co-infection were respectively 2.5% and 1.5%. This co-infection rate is twice the 0.74% rate recently found among urban pregnant women in Yaoundé [13], greater than the 0.88% reported in Burkina Faso [29], similar to the 1.3% found in Northwest Ethiopia [30], but significantly lower than the 4.2% rate reported in a study in Nigeria [31]. We found that HIV infection was highly associated with HBV infection in our study population, with HIV-infected women being more than 22 times more likely to be co-infected with HBV than HIV-uninfected ones. This finding is consistent with that of previous survey conducted among blood donors in Edéa, Cameroon [10]. This can be explained by the fact that HBV and HIV share common modes of transmission. Moreover, it has been reported that HIV/HBV co-infection facilitates HBV replication and reactivation leading to higher HBV-DNA levels and a reduced spontaneous clearance of the virus [32].

We also found that previous history of blood transfusion was associated with an increased risk of HBV infection, as reported in previous studies among pregnant women [30,33]. This result stresses the need to improve blood transfusion safety in our milieu.

Our study has some limitations. First, we used rapid diagnostic tests which are less sensitive than ELISA or PCR tests, leading to possible underestimation of the prevalence of assessed markers. Furthermore, we investigated HBV infectivity based only on HBeAg, and we did not look for anti-HBe antibodies and HBV viral load which are also important determinants of the HBV transmission. Despite these shortcomings, this study provides relevant information in a context of very limited epidemiological data on HBV infection in Cameroon, especially among pregnant women in the rural milieu.

## Conclusion

Our study suggests a high prevalence of HBV infection among pregnant women in a rural setting of the Far North region of Cameroon. History of blood transfusion and HIV infection are independently associated with the occurrence of HBV infection in this setting. The relative low prevalence of women positive to both HBsAg and HBeAg in the study population suggests that perinatal transmission of HBV might not be the major mode of HBV transmission in this area. Further studies are needed to assess thoroughly the burden and determinants of MTCT of HBV in this setting.
